# Risk of Mortality and Cardiovascular Events in Patients with Chronic Obstructive Pulmonary Disease Treated with Azithromycin, Roxithromycin, Clarithromycin and Amoxicillin in Primary and Secondary Care

**DOI:** 10.3390/biomedicines14061197

**Published:** 2026-05-25

**Authors:** Imane Achir Alispahic, Josefin Eklöf, Pradeesh Sivapalan, Alexander Ryder Jordan, Zitta Barrella Harboe, Tor Biering-Sørensen, Katja Biering Leth-Møller, Allan Linneberg, Jens-Ulrik Stæhr Jensen

**Affiliations:** 1 Department of Internal Medicine, Respiratory Medicine Section, Herlev and Gentofte Hospital, University Hospital of Copenhagen, 2900 Copenhagen, Denmark; josefin.viktoria.ekloef@regionh.dk (J.E.); alexander.ryder.jordan@regionh.dk (A.R.J.); jens.ulrik.jensen@regionh.dk (J.-U.S.J.); 2 Department of Clinical Medicine, Faculty of Health and Medical Sciences, University of Copenhagen, 2000 Copenhagen, Denmark; allan.linneberg@regionh.dk; 3 Department of Respiratory and Infectious Diseases, Copenhagen University Hospital—North Zealand, 3400 Hillerød, Denmark; zitta.barrella.harboe@regionh.dk; 4 Department of Cardiology, Gentofte University Hospital, 2900 Hellerup, Denmark; tobs@sund.ku.dk; 5 Center for Clinical Research and Prevention, Copenhagen University Hospital–Bispebjerg and Frederiksberg, 2000 Copenhagen, Denmark; katja.biering.leth-moeller@regionh.dk

**Keywords:** COPD, stroke, AMI, major adverse cardiovascular event, macrolide, clarithromycin, amoxicillin, azithromycin, roxithromycin

## Abstract

**Background:** Chronic obstructive pulmonary disease (COPD) is a progressive respiratory condition where many patients are given antibiotics like amoxicillin and macrolides (clarithromycin, azithromycin, roxithromycin) for bacterial infections. Recent concerns about clarithromycin’s potential link to cardiovascular events have arisen, despite its effectiveness against respiratory pathogens. This study aims to compare the cardiovascular risk of macrolide antibiotics versus amoxicillin in suspected COPD patients. **Method:** We used the Danish National Health Service Prescription Database (DNHSP) to identify COPD patients and their use of antibiotics. The included COPD patients were divided into four groups: amoxicillin users, roxithromycin users, clarithromycin users and azithromycin users. Data from multiple registries were merged to track hospitalizations, causes of death, and major adverse cardiovascular events (MACEs) as the primary endpoint. Patients were followed for a 3-year period. We applied adjusted Cox regression and sensitivity analyses with IPTW and IPCW to address confounders and censoring. **Results:** Our study involved 45,869 patients who were prescribed a long-acting muscarinic antagonist, over the age of 40 years old and who received one of the following antibiotics: amoxicillin, azithromycin, clarithromycin, or roxithromycin. No increased risk of MACEs was observed in macrolide-treated patients compared to those treated with amoxicillin (azithromycin: HR 0.97: 95% CI 0.83–1.13 *p* = 0.69, clarithromycin: HR 1.06 95% CI 0.87–1.28 *p* = 0.57, roxithromycin: HR 1.04 95% CI 0.91–1.18 *p* = 0.60), as confirmed by the sensitivity analysis (azithromycin: HR 0.95 95% CI 0.82–1.11 *p* = 0.52, clarithromycin: HR 1.05 95% CI 0.87–1.27 *p* = 0.60, roxithromycin: HR 1.05 95% CI 0.92–1.19 *p* = 0.48). Similarly, hazard ratios for all-cause mortality and cardiovascular death among the antibiotic groups showed no significant statistical differences. **Conclusions:** These findings suggest that there is no difference in the risk of MACEs, all-cause mortality, or cardiovascular death between the amoxicillin group and the macrolide group in a large and unselected population of COPD patients.

## 1. Introduction

Chronic obstructive pulmonary disease (COPD) is a progressive and debilitating respiratory condition characterized by persistent airflow limitation and lung inflammation. It affects approximately 300–400 million people [1] worldwide, causing substantial morbidity and mortality. Exacerbations, which lead to the deterioration of symptoms, lung function, and overall quality of life, are common among COPD patients [2].

The management of COPD involves various pharmacological treatment options, including bronchodilators, inhaled corticosteroids, and antibiotics. Antibiotics are frequently administered during exacerbations. In Denmark, amoxicillin, a beta-lactam antibiotic from the penicillin class, is commonly prescribed as the initial choice for treating COPD exacerbations [3,4].

COPD patients are at risk of having bacterial infection such as with Chlamydia pneumoniae, mycoplasma pneumonia or legionella pneumonia [5]. In such cases, macrolides like clarithromycin, azithromycin, and roxithromycin are commonly used. Macrolides are active against a range of respiratory pathogens such as pneumococci, Moraxella, and often Staphylococcus aureus, as well as Mycoplasma pneumoniae [6]. From the theory that intravascular Chlamydia infection might play a role in atherosclerosis [6] a large-scale RCT to test a macrolide therapy using clarithromycin has been conducted [7]. However, this trial indicated potential harm related to Clarithromycin in terms of myocardial infarction. The safety of other macrolides in terms of cardiovascular event risk has not been evaluated. Patients with COPD frequently undergo multiple treatments with various macrolides, including clarithromycin, roxithromycin, and azithromycin [5,6,7,8,9].

Another critical aspect to consider is the pathogenesis of atherosclerosis, which signifies the onset of a sequence that may culminate in a cardiovascular ischemic incident. This condition is marked by persistent inflammation in the arterial walls, primarily fueled by the accumulation of lipids. The involvement of both innate and adaptive immune systems is important in the evolution and advancement of this disease [8,9]. Atherosclerosis has a profound impact on the arterial structure and functionality, markedly elevating the risk of severe cardiovascular complications.

In the context of patients with COPD, the situation becomes more complex. These individuals are particularly susceptible to inflammation, often exacerbated by recurrent episodes of pneumonia or acute exacerbations. This constant state of inflammation can extend to the arterial walls, predisposing COPD patients to atherosclerosis, which might remain undetected in many cases.

Given this backdrop, the insights provided by studies such as CLARICOR [10], which highlight the potential harm of macrolides, become invaluable. These findings underscore the necessity of exploring whether similar therapeutic strategies could also be harmful for COPD patients. Such research is essential to enhance our understanding and management of the interconnectedness between chronic pulmonary conditions and cardiovascular health. The objective of this study is to determine whether patients with COPD, regardless of whether they are followed at a respiratory outpatient department or by general practitioners, face an increased risk of cardiovascular events following the use of azithromycin, clarithromycin, and roxithromycin compared to amoxicillin. By assessing the cardiovascular safety of these antibiotics, we can gain a better understanding of the risk–benefit profile of different macrolides in the management of COPD.

## 2. Methods

### 2.1. Design

We conducted a registry-based observational cohort study in the Danish population examining the risk of major adverse cardiovascular events (MACEs) for three years following antibiotic exposure. We compared patients treated with amoxicillin to patients treated with azithromycin, clarithromycin or roxithromycin.

### 2.2. Population

Danish citizens with suspected COPD, defined as patients aged 40 years or older with a reimbursed prescription of long-acting muscarinic antagonist (LAMA), were eligible for inclusion within 365 days prior to and following collection of a prescription of LAMA (ATC = RO3BB) or LAMA in combination with inhaled corticosteroids or inhaled long-acting beta 2 agonist (R03AL) between 1994 (the start of Danish National Health Service Prescription Database (DNHSP)) and 31 December 2018. Patients were included upon collection of a prescription for any of the included antibiotics; amoxicillin (ATC = J01CA04), azithromycin (ATC = J01FA10), clarithromycin (ATC = J01FA09), or roxithromycin (ATC = J01FA06). Collected prescriptions are recorded in DNHSP [11]. The date of antibiotic collection was defined as the index date. Patients were excluded if they had been diagnosed in the Danish National Patient Registry (DNPR) [12] with cancer other than basal cell carcinoma within 10 years prior to inclusion or emigrated from Denmark during the study period. The DNPR is a nationwide registry with mandatory reporting for all inpatient and outpatient hospital contacts classified according to the International Classification of Diseases 10th Revision (ICD-10). DNPR holds information on all admissions to Danish hospitals since 1977 and all hospital outpatient clinic visits since 1995. The Danish cause of death registry was used to identify cardiovascular death.

### 2.3. Outcome

The primary outcome was MACE defined as hospital admission with stroke, acute myocardial infarction (AMI) or cardiovascular death. The secondary outcome measures were all-cause mortality and cardiovascular death. Hospital admissions were defined based on ICD-10 codes using DNPR. Stroke included both ischemic and hemorrhagic subtypes, consistent with standard registry-based cardiovascular outcome definitions. Cardiovascular death and all-cause mortality was defined based on the Danish registry of causes of death [13].

### 2.4. Statistical Analysis

Descriptive analysis of the baseline characteristics, including both categorical and continuous variables, were performed using the chi-square test and Wilcoxon test, respectively, as all continuous variables were graphically assessed to be non-normally distributed. The primary and secondary outcomes were analyzed using Cox proportional hazard regression, adjusting for the suspected confounding variables: sex, age, prescriptions for acetylsalicylic acid in the year preceding baseline, prescriptions for non-vitamin K antagonist oral anticoagulants (NOAC) in the year preceding baseline, and specific comorbidities (including stroke, acute myocardial infarction, heart failure, diabetes mellitus with or without complications, and peripheral vascular disease). Comorbidities were identified using ICD-10 codes from DNPR. Heart failure encompassed both reduced ejection fraction and heart failure with preserved ejection fraction.

Patients were right censored upon end of follow-up, death, or collection of a prescription of another of the four antibiotics included in the study. For all analyses, amoxicillin was used as the reference.

In a sensitivity analysis, inverse probability of treatment weighting (IPTW) in combination with inverse propensity of censoring weighting (IPCW) [14,15] was utilized to balance confounders within the four groups and to mitigate dependent censoring. IPTW weights were calculated using multinomial logistic regression of the antibiotic group based on the covariates from the adjusted analysis. Average treatment effect (ATE) weights were calculated as the inverse probability of receiving the antibiotic treatment each patient received. IPCW weights were calculated in a similar manner by estimating the propensity towards being censored based on the same covariates as when estimating IPTW weights. Cox proportional hazard regression assumes independent censoring, and it is likely not correct to assume that risk of death or change of antibiotic is independent of the type of antibiotic received. IPCW accounts for this by balancing confounders according to likelihood of censoring. In the analysis, IPTW and IPCW weights were combined.

*p*-values less than 0.05 were considered statistically significant. All statistical analyses were performed using SAS version 9.4, SAS Institute (Cary, NC, USA), and R version 3.0, R foundation for Statistical Computing (Vienna, Austria).

## 3. Results

**Figure 1:**
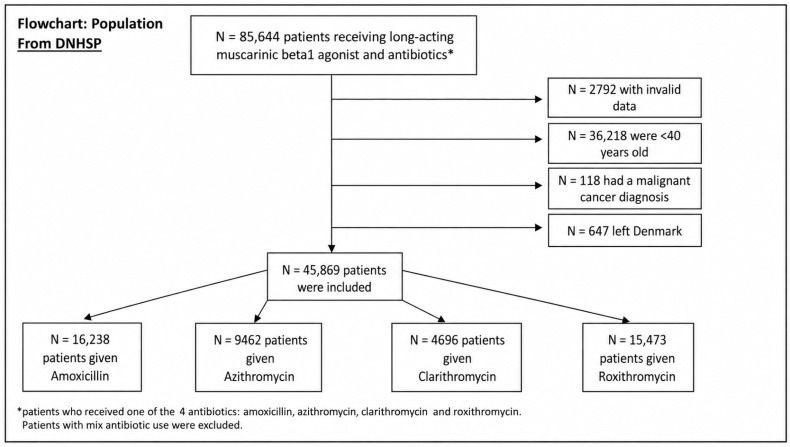
Flowchart of included patients.

We identified 45,869 patients with suspected COPD (Figure 1). There were 16,238 (35.40%) patients in the amoxicillin group, 9462 (20.63%) in the azithromycin group, 4696 (10.24%) in the clarithromycin group and 15,473 (33.73%) in the roxithromycin group. Baseline characteristics are summarized in Table 1.

Females made up roughly 52% to 53% of each group, and the median age across groups was around 57–58 years. Notable medical conditions and treatments included acetylsalicylic acid usage (about 6% across groups) and NOAC (generally below 1%). The prevalence of conditions like peripheral vascular disease, ischemic heart disease, heart failure, stroke, and diabetes (with or without complications) was below 0.3% for all groups. 

### 3.1. Primary Outcome

**Figure 2:**
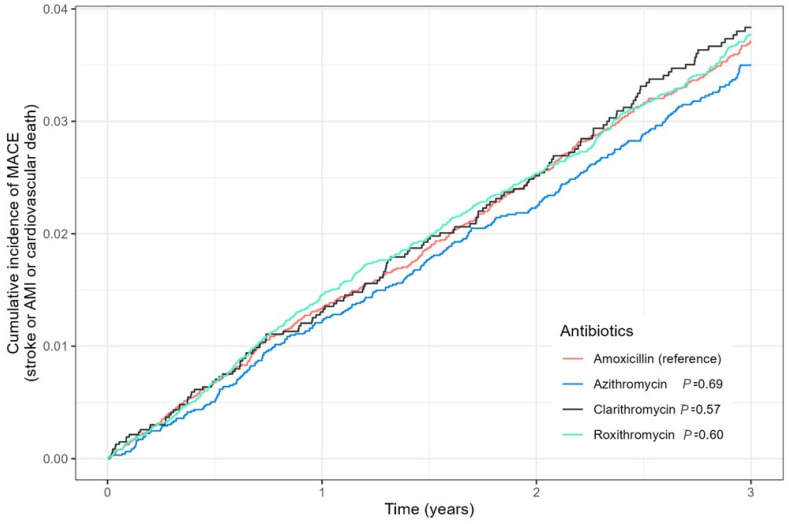
Cumulative incidence of major adverse cardiovascular events (MACEs), with non-cardiovascular death and antibiotic switch as competing events in patients with suspected COPD.

We did not find an increased risk of MACEs in macrolide-treated patients. The hazard ratios for azithromycin, clarithromycin, and roxithromycin were 0.97 (95% CI 0.83–1.13), 1.06 (95% CI 0.87–1.28), and 1.04 (95% CI 0.91–1.18), respectively (Table 2 and Figure 2), when compared to amoxicillin, and none of these hazard ratios were statistically significant. We also did a sensitivity analysis by using the combination of IPTW and IPCW, which confirmed our main findings; azithromycin HR = 0.95, 95% CI = 0.82–1.11 *p* value = 0.52: clarithromycin HR = 1.05, 95% CI = 0.87–1.27, *p* value = 0.60 and roxithromycin HR = 1.05, 95% CI 0.92–1.19, *p* value = 0.48 (Table 2).

All-cause death and subsequent antibiotics use constitute notable competing risks. For this reason, we conducted a Fine–Gray analysis, which further confirmed our main findings; azithromycin HR = 1.00, 95% CI = 0.86–1.17 *p* value = 0.97: clarithromycin HR = 1.09, 95% CI = 0.90–1.31, *p* value = 0.38 and roxithromycin HR = 1.08, 95% CI 0.95–1.24, *p* value = 0.23. Further, the lack of association remained when adjusting for use of statins in the year prior to index date.

As a post hoc analysis, we also added the number of prescriptions (one, two, three or more) into the model as a time-varying covariate, similar to what was done in Mosholder et al.’s study [16]. We found that having two or three prescriptions was associated with an increased risk of MACEs compared to no prescriptions (for two prescriptions: HR= 1.42, *p*= 0.0008 and 95% CI 1.16–1.75; for three prescriptions: HR 1.45, *p* = 0.019 95% CI 1.06–1.99), but this was independent of the type of antibiotic (p for interaction = 0.52).

### 3.2. Secondary Outcome

**Figure 3:**
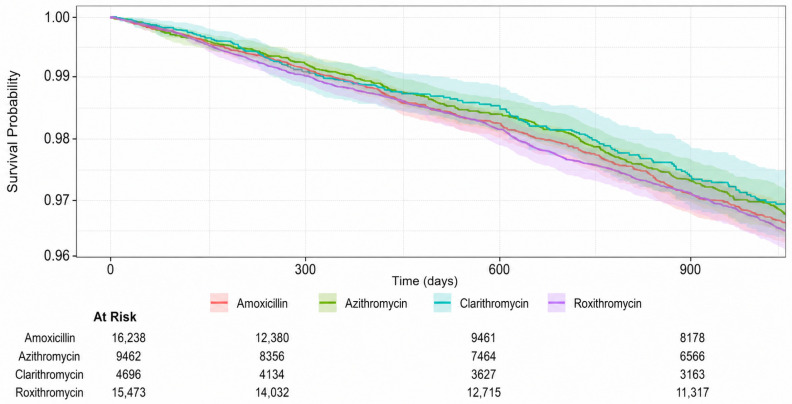
Kaplan–Meier curves showing probability of survival as well as the number of patients at risk at each time point.

We did not find any differences between the antibiotic treatment groups regarding all-cause mortality within 3 years of follow-up. Hazard ratios for azithromycin, clarithromycin, and roxithromycin, in comparison to amoxicillin, were 1.05 (95% CI 0.90–1.23), 0.95 (95% CI 0.77–1.17), and 1.08 (95% CI 0.95–1.24), respectively (Table 3). None of these hazard ratios reached statistical significance, indicating that the risk of all-cause mortality was not significantly different between these groups. The probability of survival as well as the number of patients at risk at each time point is shown in Figure 3.

Evaluating the risk of cardiovascular death did not reveal any differences between groups. Hazard ratios for azithromycin, clarithromycin, and roxithromycin when compared to amoxicillin were 1.13, 1.08, and 1.05, respectively (Table 3). We did a sensitivity analysis for the secondary endpoints by using IPTW and IPCW. The were no differences between the groups (for all-cause mortality, azithromycin HR =1.00, 95% CI 0.85–1.72, *p* value = 0.98; clarithromycin HR = 0.93, 95% CI 0.75–1.14, *p* value = 0.47 and roxithromycin HR = 1.09, 95% CI 0.95–1.25, *p* value = 0.23 and for cardiovascular death, azithromycin HR = 1.10, 95% CI 0.82–1.48, *p* value = 0.53; clarithromycin HR =1.09, 95% CI 0.75–1.58, *p* value = 0.66 and roxithromycin HR = 1.08, 95% CI 0.83–1.41, *p* value = 0.55) (Table 3 and Table 4).

Overall, based on the adjusted and IPTW combined with IPCW analyses, we did not find differences in the risk of MACEs, all-cause mortality, or cardiovascular death between the amoxicillin group and the groups treated with azithromycin, clarithromycin, and roxithromycin in the cohort.

## 4. Discussion

In our cohort analysis of 45,870 patients with suspected COPD we identified no statistically significant differences in the risk of MACEs between those treated with amoxicillin and those receiving either of the macrolides, clarithromycin, azithromycin or roxithromycin. Both adjusted Cox regression and IPTW + IPCW analysis confirmed these findings. Similarly, no differences were noted in all-cause mortality or cardiovascular mortality among these groups within the cohort.

In the ongoing discussion regarding the cardiovascular safety of macrolide antibiotics, our study adds a critical dimension by examining the implications in a population of patients managed with LAMA in a primary care setting and we consider other macrolides such as azithromycin and roxithromycin instead of just clarithromycin. This discussion covers various studies, such as randomized controlled trials and retrospective analyses, that have given mixed results on the cardiovascular outcomes linked to macrolide use [2,6,16,17,18,19,20,21,22,23,24,25,26,27,28,29,30,31,32,33,34,35,36,37,38].

Contrasting with previous findings, such as those from the CLARICOR trial [7,22,30] which indicated an increased risk of cardiovascular mortality with clarithromycin use, our study shows no statistically significant difference in the incidence of MACE when comparing clarithromycin, azithromycin, or roxithromycin to the first-choice antibiotic in suspected COPD patients, namely amoxicillin. CLARICOR compared clarithromycin to a placebo among patients with known ischemic heart disease and a documented high risk of MACEs, whereas we compared patients suspected to have COPD, using an active comparator. Another study [37] shows that azithromycin does not increase the risk of heart-related deaths compared to Penicillin V, suggesting that azithromycin is as safe as other common antibiotics for the heart. Furthermore, in another study [30] the difference between the groups seems to disappear the longer the patients were followed. In a randomized trial conducted in 2003 [33], short-term treatment with azithromycin did not reduce the number of recurring ischemic events, nor did it increase the probability of experiencing one. They included patients admitted with either unstable angina or acute myocardial infarction over the age of 18. These findings align with ours.

In a retrospective study, Mosholder and colleagues [16] compared clarithromycin vs. doxycycline and clarithromycin vs. erythromycin in the first cohort they examined. The patients were included from a UK primary care database of medical records. The patients were aged 40–85 years. They observed a statistically, significantly higher risk of death from any cause among clarithromycin users. However, this risk was less pronounced when they specifically examined stroke and acute myocardial infarction (AMI) using an IPTW analysis. Notably, their patients had a higher prevalence of heart disease, in contrast to our cohort, where the prevalence of heart conditions was low. However, as part of a post hoc analysis, we incorporated the number of prescriptions (1, 2, 3 or more) into the model as a time-varying covariate, following a similar approach used in the study by Mosholder et al. [16]. Our findings indicated that having two or three prescriptions was associated with an increased risk of MACE, which aligns with their results. However, this association is most likely explained by confounding by disease severity rather than a causal effect of antibiotic exposure.

The Tennessee Medicaid study [39], which investigated the association between clarithromycin use and sudden cardiac death, identified an association with cardiac death during the period when clarithromycin was being used. However, this risk disappeared once the use of clarithromycin was discontinued.

Furthermore, while some observational studies have suggested that macrolides could improve short-term survival rates for patients with community-acquired pneumonia [36], our findings did not demonstrate a different mortality rate when macrolides were prescribed in an outpatient context for suspected COPD. This indicates a potential difference in the impact of macrolides based on patient settings and disease severity.

Additionally, our investigation diverges from studies such as those by Schembri et al. [36], who reported an augmented risk of cardiovascular events with clarithromycin in hospitalized patients. Our primary care and outpatient-focused research, with a less critically ill cohort, might explain the discrepancy in cardiovascular outcomes.

Despite diverging findings regarding the association of macrolides with cardiovascular event risks, there appears to be a tendency for individuals with a higher baseline prevalence of cardiovascular diseases to be at an increased risk for future cardiovascular events.

Our study has several strengths. We used DNPR, which has complete data on all in- and outpatient visits to Danish hospitals for all Danish citizens. We also carefully sorted patients into four separate antibiotic groups, using DNHSP, making sure they did not overlap in treatment. One of our main methods, IPTW, helped us ensure that all groups were similar at the start, thus attempting to avoid bias by indication even though every method has its limitation. Importantly, this study is to our knowledge the first comparing amoxicillin to three other antibiotics in patients with suspected COPD, using an active comparator. Earlier studies considered using an antibiotic versus not using any.

The term “suspected COPD” is typically used when there are initial signs or symptoms that suggest COPD, but a definitive diagnosis has not yet been made. It implies that COPD is one of several possible diagnoses under consideration, and further diagnostic testing is necessary to confirm or rule out the disease. This is the rationale behind our choice to define our population using these criteria. However, there are some limitations. One of the reasons for our interest in examining patients with suspected COPD is that we have previously conducted a similar study focusing on patients with confirmed COPD [40]. In that earlier study, we specifically looked at individuals who were being followed in an outpatient setting, where their diagnosis was well established, and their condition was being actively managed. Following this, our attention shifted to patients in primary care settings, including those who may be in the earlier stages of COPD. This approach allowed us to explore a broader population, including patients whose COPD may not yet have been formally diagnosed or confirmed. By doing so, we aimed to capture a wider spectrum of disease severity and clinical presentations, which may also include individuals with overlapping conditions, such as asthma. As a consequence of this broader inclusion, it is possible that some asthma patients were inadvertently included in the study. Furthermore, this may have resulted in exclusion of individuals with milder COPD who were managed solely with beta-2 agonist therapy. However, this broader scope was intentional, as we wanted to investigate the effects of treatment in a more diverse group of patients.

We have no information about the exact indication for the antibiotics prescribed, but based on Danish prescription guidelines, we expect that the included antibiotics are most often used for lung infections, especially in this age group. One of the main limitations of our study is its retrospective design, which introduces the possibility of selection bias. Furthermore, international drug safety agencies have issued warnings regarding the cardiovascular risks associated with preexisting cardiovascular conditions. These alerts could have influenced prescribing practices and potentially impacted the risk of MACEs in both macrolide and non-macrolide users. As a result, the findings may be influenced by unmeasured confounders related to preexisting cardiovascular conditions, and caution is warranted in interpreting the results.

Another potential limitation of this study is that cardiovascular complications related to prior cancer therapies may persist beyond the applied 10-year exclusion window, particularly among childhood cancer survivors. Although extending the exclusion period or excluding all patients with any prior cancer history could potentially reduce residual confounding, such an approach could potentially decrease cohort size, limit external validity, and increase the risk of selection bias. Additionally, the lack of information regarding lipid levels may have influenced the cardiovascular outcomes in the study population. Evaluating the potential modifying effect of such measurements would have been of interest and should be explored in future studies. Furthermore, the present study has a relatively short follow-up period of 3 years, which may be insufficient to capture long-term atherosclerotic progression and related cardiovascular outcomes. Furthermore, arrhythmic outcomes such as atrial fibrillation were not evaluated separately, despite the known association between macrolides, electrophysiological disturbances, and increased stroke risk in COPD patients. Further studies are warranted to explore these associations in greater detail. Finally, Denmark is classified as a low-cardiovascular-risk country according to the SCORE2 framework, which may limit the generalizability of the findings to populations in moderate- or high-risk cardiovascular settings.

Comparing our results with other studies is difficult, especially when those studies looked at different types of patients (such as younger patients and patients with heart diseases). Lastly, there might still be residual confounding; however, we consider this less likely, since normally, second-choice drugs like macrolides for infections in COPD would be linked to a higher burden of comorbidity, and thus MACEs, than first-choice drugs.

## 5. Conclusions

We did not find any indication of a higher risk of MACEs among suspected COPD patients who had different macrolides prescribed, as compared to amoxicillin. We had complete follow-up and precise information on collected prescriptions and our results were robust to sensitivity analyses. As such, our results do not suggest changes in prescription patterns for any specific macrolide.

## Figures and Tables

**Table 1:** Baseline characteristics.

	All	Amoxicillin	Azithromycin	Clarithromycin	Roxithromycin
**Number of subjects, n (%)**	45,869 (100.0)	16,238 (35.40)	9462 (20.63)	4696 (10.24)	15,473 (33.73)
**Females, n (%)**	24,113 (52.57)	8556 (52.69)	5010 (52.95)	2499 (53.22)	8048 (52.01)
**Age, median**	57.87 (49–69)	58.21 (49–69)	57.83 (49–69)	57.91 (49–69)	57.79 (49–68)
**Acetylsalicylic acid, n (%)**	2959 (6.45)	1015 (6.25)	586 (6.19)	323 (6.88)	1035 (6.69)
**Non-vitamin K oral anticoagulants, n (%)**	333(2.77)	164 (1.01)	51 (0.54)	31 (0.66)	87 (0.56)
**Peripheral vascular disease, n (%)**	80 (0.17)	26 (0.16)	16 (0.17)	7 (0.15)	31 (0.20)
**Ischemic heart disease, n (%)**	66 (0.14)	27 (0.17)	13 (0.14)	8 (0.17)	26 (0.17)
**Heart failure, n (%)**	80 (0.17)	30 (0.18)	18 (0.19)	4 (0.09)	28 (0.18)
**Diabetes, n (%)**	93 (0.28)	37 (0.23)	28 (0.29)	13 (0.28)	15 (0.33)
**Stroke, n (%)**	66 (0.14)	27 (0.17)	13 (0.14)	8 (0.17)	18 (0.12)

**Table 2:** Primary outcome: Risk of MACEs comparing the amoxicillin group to the azithromycin, clarithromycin and roxithromycin group for patients with suspected COPD (adjusted and IPTW/IPCW *).

		Adjusted			IPTW and IPCW	
Treatment Groups	Hazard Ratio	95% Confidential Interval	*p*-Value	Hazard Ratio	95% Confidential Interval	*p*-Value
Azithromycin	0.97	0.83–1.13	0.69	0.95	0.82–1.11	0.52
Clarithromycin	1.06	0.87–1.28	0.57	1.05	0.87–1.27	0.60
Roxithromycin	1.04	0.91–1.18	0.60	1.05	0.92–1.19	0.48
Amoxicillin	ref.	ref.	ref.	1.00	ref.	ref.

* IPTW: inverse probability treatment weighting; IPCW: inverse probability censuring weighting.

**Table 3:** Secondary outcome: Risk of all-cause mortality and cardiovascular death comparing the amoxicillin group to the clarithromycin, azithromycin and roxithromycin group from patients with suspected COPD (adjusted and IPTW/IPCW *).

	All-Cause Mortality						Cardiovascular Death					
		Adjusted		IPTW and IPCW				Adjusted		IPTW and IPCW		
Treatment Groups	Hazard Ratio	95% Confidential Interval	*p*-Value	Hazard Ratio	95% Confidential Interval	*p*-Value	Hazard Ratio	95% Confidential Interval	*p*-Value	Hazard Ratio	95% Confidential Interval	*p*-Value
Azithromycin	1.05	0.90–1.23	0.55	1.00	0.85–1.72	0.98	1.13	0.83–1.53	0.44	1.10	0.82–1.48	0.53
Clarithromycin	0.95	0.77–1.17	0.61	0.93	0.75–1.14	0.47	1.08	0.73–1.60	0.69	1.09	0.75–1.58	0.66
Roxithromycin	1.08	0.95–1.24	0.25	1.09	0.95–1.25	0.23	1.05	0.80–1.38	0.71	1.08	0.83–1.41	0.55
Amoxicillin	ref.	ref.	ref.	ref.	ref.	ref.	ref.	ref.	ref.	ref.	ref.	ref.

* IPTW: inverse probability treatment weighting; IPCW: inverse probability censuring weighting.

**Table 4:** Number of events for each endpoint for each antibiotic.

Antibiotic Groups	No Events	Stroke	AMI	Cardiovascular Death	Cause of Mortality Other Than Cardiovascular Death	Antibiotic Switch ^*^	Total
	N (%)	N (%)	N (%)	N (%)	N (%)	N (%)	N (%)
**Amoxicillin**	13,890 (85.54)	248 (1.53)	81 (0.50)	71 (0.44)	254 (1.56)	1694 (10.43)	16,238 (100)
**Azithromycin**	8244 (87.13)	145 (1.53)	67 (0.71)	56 (0.59)	178 (1.88)	772 (8.16)	9462 (100)
**Clarithromycin**	3997 (85.11)	83 (1.77)	32 (0.68)	29 (0.62)	78 (1.66)	477 (10.16)	4696 (100)
**Roxithromycin**	13,653 (88.24)	287 (1.85)	112 (0.72)	93 (0.60)	329 (2.13)	999 (6.46)	15,473 (100)

* number of patients who switched from one antibiotic to another.

## Data Availability

The data supporting the findings of this study were obtained from the Danish Health Data Authority and are subject to restrictions regarding their availability, as they were accessed under license for the present study. These data are not publicly available but may be obtained from the Danish Health Data Authority upon application and approval by the authority.
